# Exploring the experiences of being an ethnic minority student within undergraduate nurse education: a qualitative study

**DOI:** 10.1186/s12912-019-0389-0

**Published:** 2019-12-05

**Authors:** Sylvi Monika Flateland, Maxine Pryce-Miller, Anne Valen-Sendstad Skisland, Anne Flaatten Tønsberg, Ulrika Söderhamn

**Affiliations:** 1 Faculty of Health and Sport Sciences, University of Agder, Post Box 422, 4604 Kristiansand, Norway; 2 University of the West England, Glenside Campus, Faculty of Allied Health, Blackberry Hill, Bristol, UK

**Keywords:** Bachelor’s degree in nursing, Content analysis, Culture, Isolation, Nursing students

## Abstract

**Background:**

Students studying in a country where another language is spoken face multiple challenges including their ability to fully integrate with peers and academic pressures in trying to obtain an undergraduate nursing degree. The aim of the study was to explore the lived experiences of students, from varying cultural and ethnic backgrounds, undertaking an undergraduate nursing degree.

**Methods:**

The study adopted a qualitative design and eight individual semi-structured interviews were conducted. The interviews were analysed using manifest content analysis according to Graneheim and Lundman.

**Results:**

Students reported feelings of isolation and the lack of opportunities to integrate with native students within academia and practice. The need for personal support was a crucial factor that was independent of gender and students reported challenges related to both language and culture during the programme.

**Conclusions:**

Suggestions arising from this study includes appropriate support systems within academia and practice. It is imperative that universities and practice settings promote and integrate cultural awareness within academia and practice in meeting the needs of students and providing culturally appropriate nursing care, thereby providing opportunities for all students to become competent and professional practitioners.

## Background

Europe is becoming increasingly multi-cultural and with globalisation, there is an increasing number of students from multiple ethnic and cultural backgrounds studying different courses within higher education. A common assumption in higher education is that academic integration of international students, that is the extent to which students adapt to the academic way-of-life [1], is not well-aligned. A large number of studies have addressed student retention or persistence in higher education. For example, the interaction student attrition model of Tinto [1, 2] considers that students have a variety of educational experiences, skills and competencies, family and community backgrounds and values before entering into higher education. At an individual and social level, this will influence how a student integrate into their studies at universities. Tinto [2] identified that students need to persist in their education to graduate, demonstrating academic integration and also need to actively participate in student culture within and outside of the learning environment.

This paper has a focus on undergraduate nurse education in Norway, where students from an international background study with native students and Norwegian is their second language. The literature indicates that students from an ethnic minority and international background face challenges in their encounters within undergraduate nursing educational systems [3–9]. Challenges related to social expectations are some of the central factors that may influence how international students may feel about their study situation [3–9]. Learning a new language and different ways of academic writing is difficult and may affect or impede academic performance. Coupled with that, ethnic minority students also reported facing discrimination and maintaining their cultural identity [6–8, 10, 11]. Additionally, these particular students may also face a sense of belonging [9].

It is reported within the literature that ethnic minority students may face discrimination at both an institutional and individual level [9, 11, 12]. Furthermore, ethnic minority students identified encountering negative experiences in clinical practice with the use of terminology and understanding patient questions [12]. Sedgwick et al. [12] identified that ethnic minority students experienced discrimination both within the academic and practice settings and there are implications. For example, there are legal statutes to protect individuals within society, and people should not be discriminated based on their gender, religion, race, and sexuality or for having a disability [13]. The literature indicates that from a European perspective, nurse education continues to reflect the values and orientation of the Euro-centric mainstream. Therefore, the inclusion of multi-culturalism and diversity within the nursing curriculum will need to be fully realised and acknowledged.

In 2017, 22,5% of undergraduate nursing students studying at universities and university colleges in Norway, were from an international background [14]. In Norway, an undergraduate nursing programme has a duration of 3 years consisting of academic and practice hours. Students must compete on equal terms for entry onto the programme and education is delivered in Norwegian. International students must demonstrate proficiencies in the Norwegian language by undertaking and passing a test, verbal and writing and are held to the highest standards. In 2017, at one particular university, 11% of the students were from an ethnic minority background mainly Asia and Africa [14]. These students are integrated into the undergraduate nursing programme with native students.

Migration often brings many social, cultural, and psychological losses, which may cause an individual to encounter feelings of isolation when settled in another country with different cultural norms and values. Consequently, there may be difficulties faced in trying to adjust and feel a part of society. With this in mind, it is imperative that ethnic minority students are given due consideration and experience a positive teaching and learning environment to promote integration [10, 11]. Additionally, a lack of role models amongst family, friends and within the academic and practice environment is another factor that can impact ethnic minority students’ experiences, leading to feelings of vulnerability more so than other native students [5, 15]. More research is needed among this particular group of students, so that their experiences can be captured and better understood [12]. Therefore, higher education institutions have a responsibility in enabling all students, irrespective of backgrounds or cultural identity to achieve their full potential [3]. There are only a few studies that have been conducted from a Scandinavian perspective about ethnic minority undergraduate nursing students’ experiences within undergraduate nurse education. Therefore, the aim of this study was to explore the lived experiences of students from varying cultural and ethnic backgrounds undertaking an undergraduate nursing degree.

## Methods

### Design

This study adopted a qualitative design, using individual semi-structured interviews with eight ethnic minority undergraduate nursing students from this university in Norway during 2017. A qualitative approach was chosen to gain in-depth insights and further understanding into the students’ subjective experiences and social context in their natural setting [16] using a loose topic guide to allow students the freedom to elaborate and guide the discussion.

### Recruitment and sample

The inclusion criteria were undergraduate nursing students with migrant status living in Norway and undertaking an undergraduate nursing degree at one university. There is one intake of students per year and students were recruited from the third year of their study over two intakes. The intention was to recruit students from the third year in the programme who were near completion to explore differences or similarities in their journey. The rationale for including third year students was the length of time on the programme and the ability to provide a wider perspective. Students who fulfilled the inclusion criteria (*n* = 11) were invited by the coordinators of the nursing bachelor programme to participate in the study, which is purposive sampling. Information was provided about the study by the coordinators and 8 of the 11 students contacted agreed to participate in the study. Students were informed that they were not obligated to participate in the study to prevent co-coercion. Participants were born in different countries ranging from Asia and Africa, respectively. Participants had been living in Norway from 6 to 25 years, respectively, and consisted of five women and three men aged between 22 and 37 and had all Norwegian citizenship. All participants were considered as mature students given the age range.

### Data collection

Eight individual semi-structured interviews lasting 25 to 45 min were conducted by the first author. The interviews were conducted in a private room at the local university campus and audio recorded with consent. Employing face-to-face dialogue and a loose topic guide, participants were free to move the interview in any direction to best describe their experiences of being an ethnic minority student within the academic and practice environment. After obtaining demographic data including age, gender, country of birth, and length of time each individual had lived in Norway open-ended questions were then asked: “Can you tell me about your experiences of being a student in this nursing programme?” Probing questions including, “What do you mean?” and “Can you tell me a little more about …?” were asked to gain further in-sight and depth of conversation. A topic guide was used as a checklist to get the participants’ experiences elucidated around lectures, student life at campus, working in groups with other students, practice placements within the hospital and community settings. Interviews were transcribed verbatim.

### Analysis

The transcribed interviews were analysed using manifest qualitative content analysis according to Graneheim and Lundman [17]. The steps in the manifest analysis were as follows: (1) individual interviews were read several times to achieve a sense of the entire picture; (2) a number of 230 meaning units were then selected (based on each participant’s experiences of being an ethnic minority student in an undergraduate nursing programme); (3) each meaning unit was condensed; (4) the condensed meaning units were abstracted and labelled with a code; and (5) a number of 30 codes derived from the data were gathered and sorted into three categories (6). Three of the authors discussed the analysis and verified the obtained codes and categories. In the analysis, the authors used their insider knowledge as nurses and nurse educators to make sense of the data. A practical example of the analysis process is displayed in Table 1 below.

**Table 1:** A practical example from the qualitative analysis

Participant	Meaning unit	Condensed meaning unit	Abstracted meaning unit/code	Categories
8	“I feel lonely. I am afraid for asking the other students.”	The student was speaking about feelings of being alone and isolated.	The student felt isolated.	Feeling isolated
4	“I feel I need a person who I could ask about everything connected to be a nurse.”	The student expressed a need of interpersonal support.	The student felt a need of interpersonal support.	Student support
1	“It is difficult for me to write, to choose right words, to find synonyms – to formulate in an adult way. I feel I have a childish language.”	The student found it difficult to write in an academic way and perceived to have a childish language.	The student had difficulties in writing and had a poorly language.	Challenges with language and culture

## Results

### Feeling isolated

Most participants identified and spoke about their experiences and feelings of isolation during their years within the nursing programme. This was significant and was linked to more engagement in group work at the university: *“I don’t have bad feelings in the classroom, but when we work in groups, I feel isolated”* (Participant 6). Participants reported that conversations in groups sometimes were conveyed in ways that they did not understand: *“I went to the group where I was placed, and we started to speak about the lesson for the day. After a brief time, one of the students said: Bye, we are meeting next Monday. I hadn’t catch what we had agreed”* (Participant 3). Even though ethnic minority students tried very hard to be a part of the group through the programme, most of them felt that they did not fully grasp the language and that the Norwegian students did not modify their language to accommodate for this.

While some of the participants experienced greater degrees of isolation, other felt less: *“In my group we are on Facebook. There I can ask them about questions I have due to this topic”* (Participant 5). Only one of the female participants expressed that her knowledge was appreciated within the group and felt valued as a member: *“They always told me that I was very clever, and they supported me and said that: We listen to you, while we do the writing work. We’re kidding in a way and I use to say that they had to cope with my orthography and that worked!”* (Participant 7).

Native students sometimes organise and hold social events in the evenings. At break times, native students get together, and some participants reported feeling like outsiders and noted that break times within academia were uncomfortable points during the day. Students reported that they had attempted to interact with native students on several occasions but found it difficult to feel a sense of belonging with native peers: *“I had decided from the beginning that I should get contact with Norwegian students, but I see that I don’t catch it”* (Participant 1). Most participants reported that after 3 years, they could not identify one native students on the programme as a friend. Additionally, those who had been in Norway since childhood had also found it difficult to make friends throughout their years at school. Each participant addressed their loneliness in their own way, by getting together as a group within the wider group of students: *“I established a group for all the students at the programme coming from another culture. We meet once in a month for exchange experiences and help each other”* (Participant 8).

### Student support

All participants reported that they did not know anyone outside the university who could support them in a largely unknown world, as represented by the university and the undergraduate nursing programme. Six of the participants reported facing difficulties in making the transition from high school to university. At the start of the programme most participants noted that some native students knew individual students beforehand, such as those from their own families or other individuals whom they could talk to about the reality of being a university student. For example, some native students were able to speak with someone who had previous experience as a nurse or had completed a degree. The participants missed this opportunity: *“Nobody in my family has higher education. I didn’t know anything about either student life at the university or being a student in practice in the Norwegian Health Care Services. I have to find it out of my own”* (Participant 5).

All participants, irrespective of gender, expressed a need for interpersonal support from nurse educators within the academic sector. Participants reported that during the duration of the nursing programme, it would have been beneficial if they had been given a mentor: *“I should wish I had someone who could guide me through the programme, a person I could ask all those stupid questions I had”* (Participant 4). Only one male participant reported having a native student that followed him up from the beginning of the programme until the final delivery of student’s bachelor’s thesis: *“I meet* [the name of the person] *at the beginning of the programme and* [the name of the person] *has helped and encouraged me and…has meant a lot for me! …always saying: You can do this!”* (Participant 2).

Participants reported student support in clinical practice as challenging, both within the hospital and community settings. They described a plethora of emotions including fear, anxiety, curiosity and happiness. However, participants recounted a number of negative encounters within the clinical area, and in particular those placed within the hospital setting. These included supervisors referring to native students by name but addressing ethnic minority students not by their names but as students. Another example reported was supervisors speaking negatively about ethnic minority students with colleagues in their presence: *“My supervisor talked about me to another nurse, while I was standing next to her. She was speaking in a way as I didn’t exist!”* (Participant 8). Some participants perceived a few supervisors in practice as being unfriendly and stated that they tried to avoid them. Conversely, some participants communicated working closely with nurse supervisors, not because they needed to, but because they felt it was a necessary step to pass clinical practice: *“I felt I had to be close to my nurse if I should pass through hospital practice”* (Participant 3). However, other expressed that they had a good relationship with their supervisors: *“From the first time it functioned in a good way between my nurse supervisor and me”* (Participant 4).

Most participants conveyed curiosity linked to starting clinical practice. They related working with patients as very interesting and narrated that it was easy to establish a good relationship with most patients. However, some participants reported a few negative encounters with patients, who requested reassignment as they did not want care delivery from an ethnic minority student: *“I had responsibility for a patient to clarify him for leaving the hospital, but the problem was that the patient didn’t want me to do that and I felt it was because of my skin colour. My supervisor was whining at me and she felt that I used more time than I should on this work”* (Participant 5).

### Challenges with language and culture

Seven participants reported academic writing as very challenging and felt they possessed rudimentary language skills. Three of them also mentioned that they had good marks in high school, but it was difficult for them to maintain the same high grades in university when compared with the grades achieved among native students: *“It is more difficult for me to get good marks than it is for them. I was very clever at high school!”* (Participant 6). Due to their language-related challenges, participants wished for more academic support for most of the compulsory written assignments in their university programme.

Participants described additional responsibilities that they had outside of university life. This ranged from taking care of family members including parents, which they thought native students did not need to do. Participants described conflicts as they had to balance the commitments of being a university student and family responsibilities and duties expected from them culturally. This led to some participants feeling torn between wanting to take care of their families and wanting to feel a similar sense of independence that was experienced by the native students: “*I have taken care for my mom and she don’t have a good health. When I use time with my fellow students, I know that she misses me and want me back home”* (Participant 7).

## Discussion

The aim of this study was to explore the lived experiences of students from varying cultural and ethnic backgrounds undertaking an undergraduate nursing degree. The findings identified that participants faced discrimination in practice and felt isolated generally. Additionally, all participants spoke of their desire to integrate and build relationships with native students. This is supported by With and Fulton [5] who reported similar findings and identified that ethnic minority students need to develop relationships with their peers throughout the course of their education. The findings from this study is a clear indication of the need for native students, nurse educators in practice and academia to give due regard to multi-culturalism and its significance in moving forward. All students can learn from each other and integration would allow for peer learning and diversity to be addressed. Given the changing nature of higher education and globalisation, there is a concerted need for systematic policies and procedures in place that values diversity, equity and student progression and not just seen as a tick box exercise.

All participants experienced isolation, despite living in Norway for a number of years. Reasons identified by participants were cultural diversity and different ways of living, as some mentioned family commitments and cultural expectations. Therefore, it is imperative that cultural diversity is acknowledged and developed within the curriculum to truly create an inclusive environment within academia and practice. This requires a consistent approach and action plan, evaluated annually to address concerns and identify best practice. Nursing values as empathy and caring is a requirement of a professional nurse and this should also relate to colleagues and this will need to be made explicit to all students irrespective of background to culture [7]. This may be one way in getting ethnic minority and native students to integrate more and reduce feelings of isolation, thereby creating a sense of belonging. This is important for delivering culturally competent nursing care within a multicultural society. Furthermore, building relationships is closely concerned with communication between people, as in previous studies by Mattila et al. [8] and Pitkäjärvi et al. [18], who found that language barriers increases students’ risk of isolation. Struggling with different accents may hinder confidence in communicating effectively and could further compound the way that ethnic minority students are perceived by native students [6]. One way in which confidence and communication can be improved amongst this particular group of students is to provide opportunities to attend an advanced language course before starting the programme. Since language difficulties will influence both the relationship and confidence of students, the use of additional language skills is vital in preparing and enhancing student learning. Participants reported that social events held outside of university and break times did not aid interaction with native students. These finding are similar to White and Fulton [5] and resulted in the ethnic minority students establishing their own group for peer support. It is evident that academia and practice will need to collaborate further in ensuring the needs of this particular group of students are identified and met. Additionally, the curriculum will need to be further developed to ensure that student attainment is comparable for ethnic minority students.

Cooperating with nurse supervisors was difficult for some participants and participants within this study alluded to facing discrimination and racism in clinical practice and being poorly treated based on their ethnicity. This was further compounded by a few patients who requested to be cared for by natives rather than an ethnic minority student. Orduňa [19] found similar results among African American students and reported that some patients requested reassignment because they did not want care delivery from that particular group of students. These findings illuminate that further cooperation between clinical practice and the university is vitally important in addressing students concerns and complaints of discrimination. Discrimination is against the law and policies should be utilised effectively to send a message that it is not acceptable and educators both within academia and practice should undertake staff development including unconscious bias training. White and Fulton [5] reported that ethnic minority students were more likely to face discrimination from nurse supervisors in clinical practice and Gardner [7] found that ethnic minority students were more likely to be excluded or overlooked. This can also be a problem for native Norwegian students. As reported by Sedgwick et al. [12], the participants in this study tried to avoid nurse supervisors they perceived as being unfriendly. This can be detrimental on student morale and performance. Students need to feel respected, encouraged and treated with dignity [6]. Inappropriate behaviour should be challenged and addressed to truly promote a more inclusive working environment for ethnic minority students. The findings of this study identified other factors that were negative for ethnic minority students including lack of peer support and a deficit of role models, which appeared to amplify the challenges they faced [5, 15, 20]. This can also be a problem for native Norwegian students. The knock-on effect for ethnic minority students is to see role models they can aspire to and to enlighten native students on becoming more diverse in their outlook. Skisland et al. [21] also found that supervisors in clinical practice requested closer cooperation with the university in improving supervision of ethnic minority students. One way to promote inclusivity is for higher education institutions and practice settings to employ individuals from diverse backgrounds, which is in keeping with globalisation and the diverse growing population within Norway.

### Implications for nursing education practice

The findings from this study have implications for undergraduate programmes in Norway from an institutional perspective in providing and approaching diversity and inclusivity in a more productive and systematic way. In moving forward, diversity training should be mandatory for staff within academia and practise. This would go some way in promoting and enhancing diversity and inclusivity. Additionally, the curriculum will need to be developed to promote inclusivity and equity, taking into account individual learning needs of students from all backgrounds. Establishing a mentoring programme or buddy system for ethnic minority students may go some way in alleviating some of the challenges identified by participants within this study. Receiving additional support from academics and peers will be invaluable in enhancing confidence and promoting integration. This is where the role of having a personal academic tutor may be invaluable in providing one-to-one support for students. Furthermore, the cooperation between universities and clinical practices should be more aligned to ensure that students are treated fairly and with respect irrespective of their cultural background.

### Strengths and limitations

The trustworthiness of the study was ensured by the fact that a) all participants were encouraged to speak freely about their experiences; b) all the steps in the analysis [17] were strictly followed; c) three of the authors participated in the coding of the data and d) the students’ voices were heard and accounted for using quotations. Moreover, the findings were linked with those from other international studies [4, 10–12, 18, 20], which bolsters the integrity of the current study. Thus, this study has demonstrated that participants experienced similar challenges as ethnic minority students’ in other western countries. Whilst the sample size is small, the findings may resonate to other undergraduate nursing programmes, but as with other qualitative research, it is up to the reader to decide on the level of transferability [16, 17].

One limitation in the present study was the sample size as only eight students were recruited. Despite the small sample size, rich data were obtained, and saturation was achieved. The topic guide was not pilot tested due to time constraints and this could be another limitation. Another limitation could be that it was difficult for participants to be completely honest with the teacher about their experiences of being a nursing student. The first author had met a few of the participants during their second year in practical studies. When information was provided about the study, students were informed that participation was voluntary. A year had elapsed when the interviews were performed, and three students chose not to participate. Further research is needed to develop a comprehensive nursing education programme where all students are given the same opportunities in becoming healthcare professionals that values diversity and inclusion. It would be useful to conduct this study in other European countries such as the United Kingdom to gain a European perspective.

## Conclusion

Most participants in the study faced many challenges with language, culture and isolation throughout the undergraduate programme in nursing as documented within the findings and discussion. The findings indicate that it is important for ethnic minority nursing students to have social support and be surrounded by a strong network and role models. It is considered particularly important that nursing educational programmes incorporate intercultural knowledge and guidance into their work with the students.

Universities will need to promote multi-culturalism for all students and staff as part of the education process, as this is significant in nursing care delivery. Further collaboration and training is required within academia and practice and is imperative in ensuring that all students get a quality education and the individual needs of ethnic minority students are met. Nurse educators in practice will need to work within the law and educate patients in preventing discrimination and hospital policies should be very clear in these matters. The number of ethnic minority students will increase as will the patient population and meeting the needs of these individuals will need to be a priority.

## Data Availability

The interview data will not be shared since the participants are guaranteed full anonymity.
